# Structure and content of the EU-IVDR: Current status and implications for pathology

**DOI:** 10.1007/s00292-022-01176-z

**Published:** 2023-02-03

**Authors:** Andy Kahles, Hannah Goldschmid, Anna-Lena Volckmar, Carolin Ploeger, Daniel Kazdal, Roland Penzel, Jan Budczies, Gisela Kempny, Marlon Kazmierczak, Christa Flechtenmacher, Gustavo Baretton, Wilko Weichert, David Horst, Frederick Klauschen, Ulrich M. Gassner, Monika Brüggemann, Michael Vogeser, Peter Schirmacher, Albrecht Stenzinger

**Affiliations:** 1 Institute of Pathology, Heidelberg University Hospital, Heidelberg, Germany; 2 Professional Association of German Pathologists, Bundesverband Deutscher Pathologen e. V., Berlin, Germany; 3 Department of Pathology, University Hospital Carl Gustav Carus, Technische Universität Dresden, Dresden, Germany; 4 Institute of Pathology, Technical University of Munich, Munich, Germany; 5 Institute of Pathology, Charité—Universitätsmedizin Berlin, Berlin, Germany; 6 Institute of Pathology, Ludwig-Maximilian University of Munich, Munich, Germany; 7 Faculty of Law, University of Augsburg, Augsburg, Germany; 8 2nd Internal Medicine Department, Hematology Lab Kiel, University Hospital Schleswig-Holstein (UKSH), Kiel, Germany; 9 Institute of Laboratory Medicine, Ludwig-Maximilian University of Munich, Munich, Germany

**Keywords:** Diagnostic reagent kits, Quality of healthcare, Government regulation, In-house production, Laboratory-developed tests

## Abstract

**Background:**

Regulation (EU) 2017/746 on in vitro diagnostic medical devices (IVDR) was passed by the European Parliament and the Council of the European Union on 5 April 2017 and came into force on 26 May 2017. A new amending regulation, which introduces a phased implementation of the IVDR with new transitional provisions for certain in vitro diagnostic medical devices (IVDs) and a later date of application of some requirements for in-house devices for healthcare facilities, was adopted on 15 December 2021.

The combined use of CE-certified IVDs (CE-IVDs), in-house IVDs (IH-IVDs), and research use only (RUO) devices are a cornerstone of diagnostics in pathology departments and crucial for optimal patient care. The IVDR not only regulates the manufacture and placement on the market of industrially manufactured IVDs, but also imposes conditions on the manufacture and use of IH-IVDs for internal use by healthcare facilities.

**Objectives:**

Our work provides an overview of the background and structure of the IVDR and identifies core areas that need to be interpreted and fleshed out in the context of the legal framework as well as expert knowledge.

**Conclusions:**

The gaps and ambiguities in the IVDR crucially require the expertise of professional societies, alliances, and individual stakeholders to successfully facilitate the implementation and use of the IVDR in pathology departments and to avoid aberrant developments.

Regulation (EU) 2017/746 on in vitro diagnostic medical devices (IVDR), fully applicable since May 26, 2022, and the associated fulfillment of the conditions pose new challenges for health institutions and thus also for diagnostic facilities such as departments of pathology. This article is intended to provide an overview on the background and framework conditions as well as to identify areas of IVDR that need to be filled in and supplemented by specialist scientific expertise to achieve successful implementation and application in laboratories.

Regulation (EU) 2017/746 on in vitro diagnostic medical devices (hereinafter: IVDR) was adopted by the European Parliament and the Council of the European Union on April 5, 2017, and entered into force on May 26, 2017 [31]. This regulation repeals the EU Directive 98/79/EC on in vitro diagnostic medical devices (abbreviated IVDD for in vitro diagnostic medical devices directive) [21], which had been in force since 1998. Once adopted, an EU regulation is a valid and a binding legislative act that all EU member states must apply by in its entirety. Therefore, in contrast to the now superseded EU Directive, it does not first have to be transposed into national law [16]. Together with the IVDD, the German Medical Devices Act (*Medizinproduktgesetz*, MPG), which serves as the German implementation of the IVDD, will thus also become invalid (for definitions of terms, see Table 1).

**Table 1:** Definitions according to IVDR 2017/746 [31]

	Term	Definition according to IVDR 2017/746	Reference
#1	**Device**	*“For the purposes of this Regulation, ****in vitro diagnostic medical devices ***(see #2)* and ****accessories for in vitro diagnostic medical devices*** (see #3)* shall hereinafter be referred to as ‘devices’.”*	Article 1 (2)
#2	**In vitro diagnostic medical device**	*“In vitro diagnostic medical device means any ****medical device*** (see #4) *which is a reagent, reagent product, calibrator, control material, kit, instrument, apparatus, piece of equipment, software or system, whether used alone or in combination, intended by the manufacturer to be used in vitro for the examination of specimens, including blood and tissue donations, derived from the human body, solely or principally for the purpose of providing information on one or more of the following:* *(a) concerning a physiological or pathological process or state;* *(b) concerning congenital physical or mental impairments;* *(c) concerning the predisposition to a medical condition or a disease;* *(d) to determine the safety and compatibility with potential recipients;* *(e) to predict treatment response or reactions;* *(f) to define or monitoring therapeutic measures.* *Specimen receptacles shall also be deemed to be in vitro diagnostic medical devices.”*	Article 2 (2)
#3	**Accessory for an in vitro diagnostic medical device**	*“Accessory for an in vitro diagnostic medical device means an article which, whilst not being itself an ****in vitro diagnostic medical device***, (see #2)* is intended by its manufacturer to be used together with one or several particular in vitro diagnostic medical device(s) to specifically enable the in vitro diagnostic medical device(s) to be used in accordance with its/their intended purpose(s) or to specifically and directly assist the medical functionality of the in vitro diagnostic medical device(s) in terms of its/their intended purpose(s);”*	Article 2 (4)
#4	**Medical device**	*“Medical device means any instrument, apparatus, appliance, software, implant, reagent, material or other article intended by the manufacturer to be used, alone or in combination, for human beings for one or more of the following specific medical purposes:* *– diagnosis, prevention, monitoring, prediction, prognosis, treatment or alleviation of disease,* *– diagnosis, monitoring, treatment, alleviation of, or compensation for, an injury or disability,* *– investigation, replacement or modification of the anatomy or of a physiological or pathological process or state,* *– providing information by means of in vitro examination of specimens derived from the human body, including organ, blood and tissue donations,* *and which does not achieve its principal intended action by pharmacological, immunological or metabolic means, in or on the human body, but which may be assisted in its function by such means.* *The following products shall also be deemed to be medical devices:* *– devices for the control or support of conception;* *– products specifically intended for the cleaning, disinfection or sterilisation of devices as referred to in Article 1 (4) and of those referred to in the first paragraph of this point.”*	Article 2 (1) Regulation (EU) 2017/745 on medical devices (MDR) [30]
#5	**Economic operator**	*“Economic operator means a manufacturer, an authorised representative, an importer or a distributor.”*	Article 2 (28)
#6	**Health institution**	*“Health institution means an organisation the primary purpose of which is the care or treatment of patients or the promotion of public health.”*	Article 2 (29)
#7	**Notified body**	*“Notified body means a conformity assessment body designated in accordance with this Regulation”; *(list see [12])	Article 2 (34)

The introduction of the IVDR is intended to reduce the risk of national differences in the interpretation of the IVDD within the EU. The original version of the IVDR provided, after a transition period of 5 years, that all requirements of the IVDR for an in vitro diagnostic medical device must be fully met as of May 26, 2022. Exemptions and transition periods for economic operators are regulated by Article 110 of the regulation. Deviating from the initial deadline definition, the European Commission—following a request by an intergroup letter of the European Parliament and a decision of the Council of Health Ministers—proposed amended transitional provisions for certain in vitro diagnostic medical devices and introduced a later start date for some requirements for in-house devices for health institutions on October 14, 2021 (Table 2; [13, 14]). This proposal was adopted on December 15, 2021 [15]. In this regard, the new amending regulation only refers to the phasing-in of the requirements and does not change any of the requirements in the original regulation.

**Table 2:** Phased introduction of EU Regulation 2017/746 on in vitro diagnostic medical devices (IVDR) [13–15]

On October 14, 2021, the European Commission proposed amended transitional provisions for certain in vitro diagnostic medical devices and a later start date for the requirements for in-house devices (IH-IVDs) due to the inability to meet the introduced changes and measures for economic operators and health institutions by the deadline due to the COVID-19 pandemic [13, 14]. On December 15, 2021, the European Parliament approved the European Commission’s draft legislation extending the deadlines for partial aspects of the IVDR [15]. One of the deciding factors for this proposal was the still very small and limiting number of notified bodies. Currently (as of January 19, 2023), the number of notified bodies is eight, distributed among five member states (3× Germany, 2× Netherlands, 1× France, 1× Slovakia, 1× Austria) [12]. These reasons put both the few notified bodies and the large number of economic operators and health institutions, which are opposed to this small number, in front of obstacles that cannot be overcome for the time being. With the gradual introduction of the IVDR, manufacturers and notified bodies will be allowed more time to demonstrate the IVDR compliance of their devices. Also determined are phased transition periods for implementing the conditions for in-house devices under Article 5 (5). For condition d), the longest transition period will be until May 26, 2028. The transition periods for conditions b), c), and e) to i) will extend to May 26, 2024. Condition a) and the requirement that in-house devices comply with the essential safety and performance requirements of Annex I and may not be manufactured on an industrial scale are exempt from the transition periods and the May 26, 2022, deadline continues to apply. However, condition (f), which requires a public declaration for compliance with the essential safety and performance requirements of Annex I, does not apply until May 26, 2024	
**CE-IVDs**	
New devices for which neither a certificate from a notified body nor a Declaration of Conformity according to Directive 98/79/EC is available	No extended transition period; May 26, 2022, applies
Class A legacy devices (without participation of notified bodies)	No extended transition period; May 26, 2022, applies
Class A legacy devices (with participation of notified bodies)	New transition period: May 26, 2027
Class B legacy devices	New transition period: May 26, 2027
Class C legacy devices	New transition period: May 26, 2026
Class D legacy devices	New transition period: May 26, 2025
**IH-IVDs—Article 5 (5)**	
Annex I + a)	No extended transition period; May 26, 2022, applies
Condition b), c), e), f), g), h), and i)	New transition period: May 26, 2024
Condition d)	New transition period: May 26, 2028
**Recital of stepwise transition periods for IH-IVDs under Article 5 (5)** [14]**:**	
*“(9) Having regard to the resources required from health institutions in the fight against the COVID-19 pandemic, those institutions should be given additional time to prepare for the specific requirements laid down in Regulation (EU) 2017/746 for the manufacture and use of devices within the same health institution (‘in-house devices’). The application of those requirements should therefore be deferred. As the health institutions will need a complete overview of CE marked in vitro diagnostic medical devices available on the market, the requirement to provide justification that the target patient group’s needs cannot be met, or cannot be met at the appropriate level of performance, by a device available on the market should not become applicable until the transitional periods laid down in this Regulation have ended.”* [14]	

## Intention

In the preamble to the IVDR, the European Parliament and the Council of the European Union provide 101 recitals for the replacement of the 1998 IVD Directive 98/79/EC (IVDD) by IVD Regulation 2017/746 (IVDR), focusing on health protection through high patient and user safety and a functioning internal market through harmonization of legislation. Primarily, the IVDR regulates the placing on the market, the provision on the single European market, and a risk-based classification of the devices according to their intended purpose and the respective resulting requirements. These regulations mainly address economic operators, i.e., manufacturers, distributors, and importers of in vitro diagnostic medical devices. However, the IVDR also concerns the use of in vitro diagnostic medical devices in health institutions. Recital 29 of the IVDR emphasizes the special importance of health institutions and the in vitro diagnostic devices they develop themselves (hereinafter IH-IVD for in-house in vitro diagnostic devices; also referred to as laboratory developed tests [LDT]). Health institutions should continue to be able to manufacture, modify, and use devices in-house to meet the specific needs of patients. However, health protection is to be increased by now stricter requirements (recital 28). For the first time, the IVDR attempts to create harmonized conditions throughout the EU and imposes several requirements on health institutions that develop and use in-house IVDs.

## Use of in vitro diagnostic medical devices in health institutions

Health institutions such as hospitals, institutes of pathology, medical laboratories, and health care centers use commercial IVDs as consumers in their diagnostic process chains (CE-certified IVDs [CE-IVDs], but also research use only [RUO] devices). They also develop, optimize, implement, and validate in-house diagnostic procedures and materials. For example, a study from a Belgian university hospital found that although almost all test results were obtained with CE-IVD-labeled procedures (98%), of the different IVD devices used, about half (47%) were developed in-house (IH-IVDs), with no commercial alternatives available for the majority (72%) [29].

**Fig. 1:**
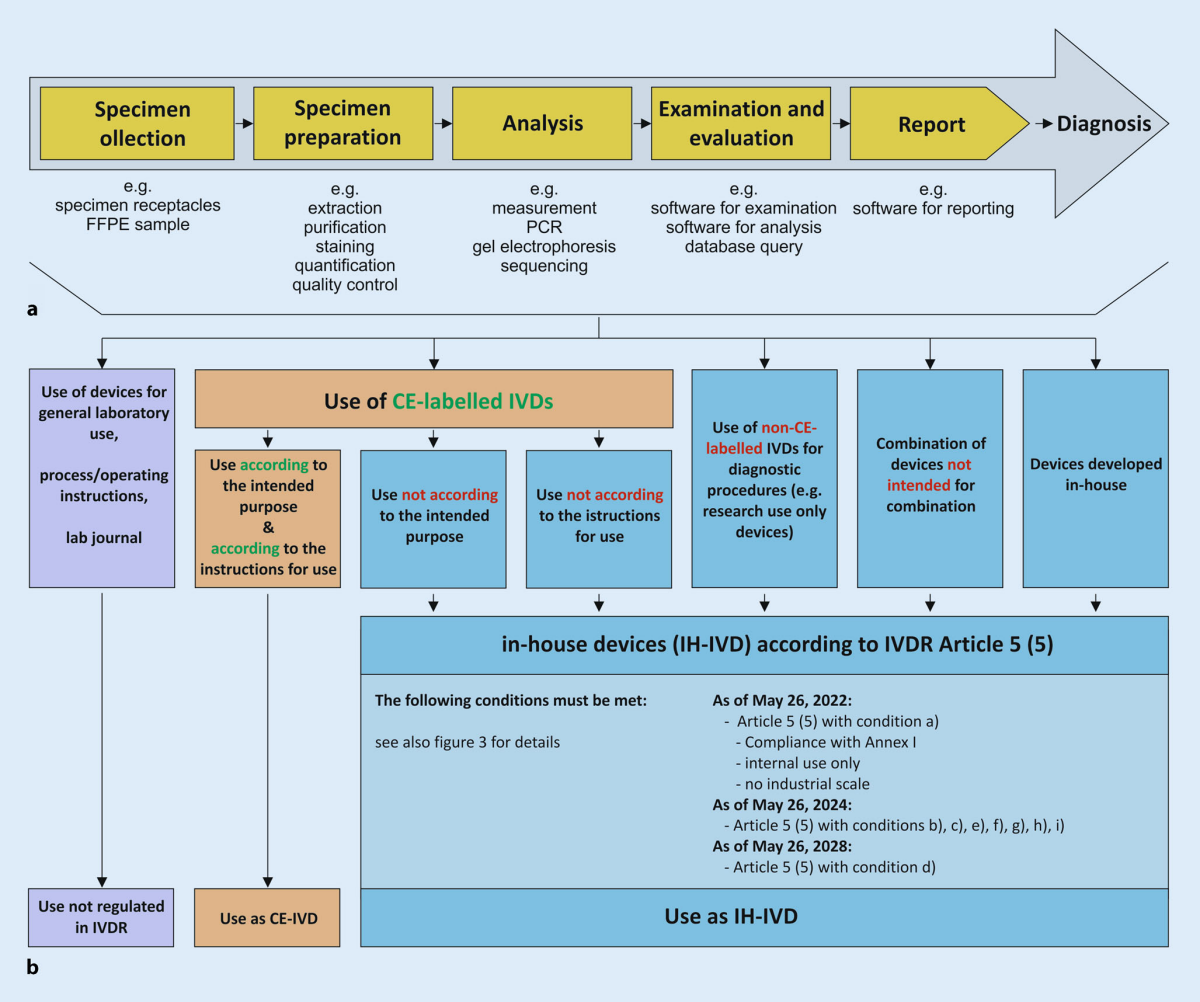
**a** Process chain in the analytics of health institutions. **b** Each link in the process chain (**a**) from specimen receipt to diagnostic findings may contain devices that can be assigned to either general laboratory use (*purple*), CE-marked in vitro diagnostic devices (*CE-IVD*, *orange*), or in-house developed IVDs (*IH-IVD*, *blue*)

**Fig. 2:**
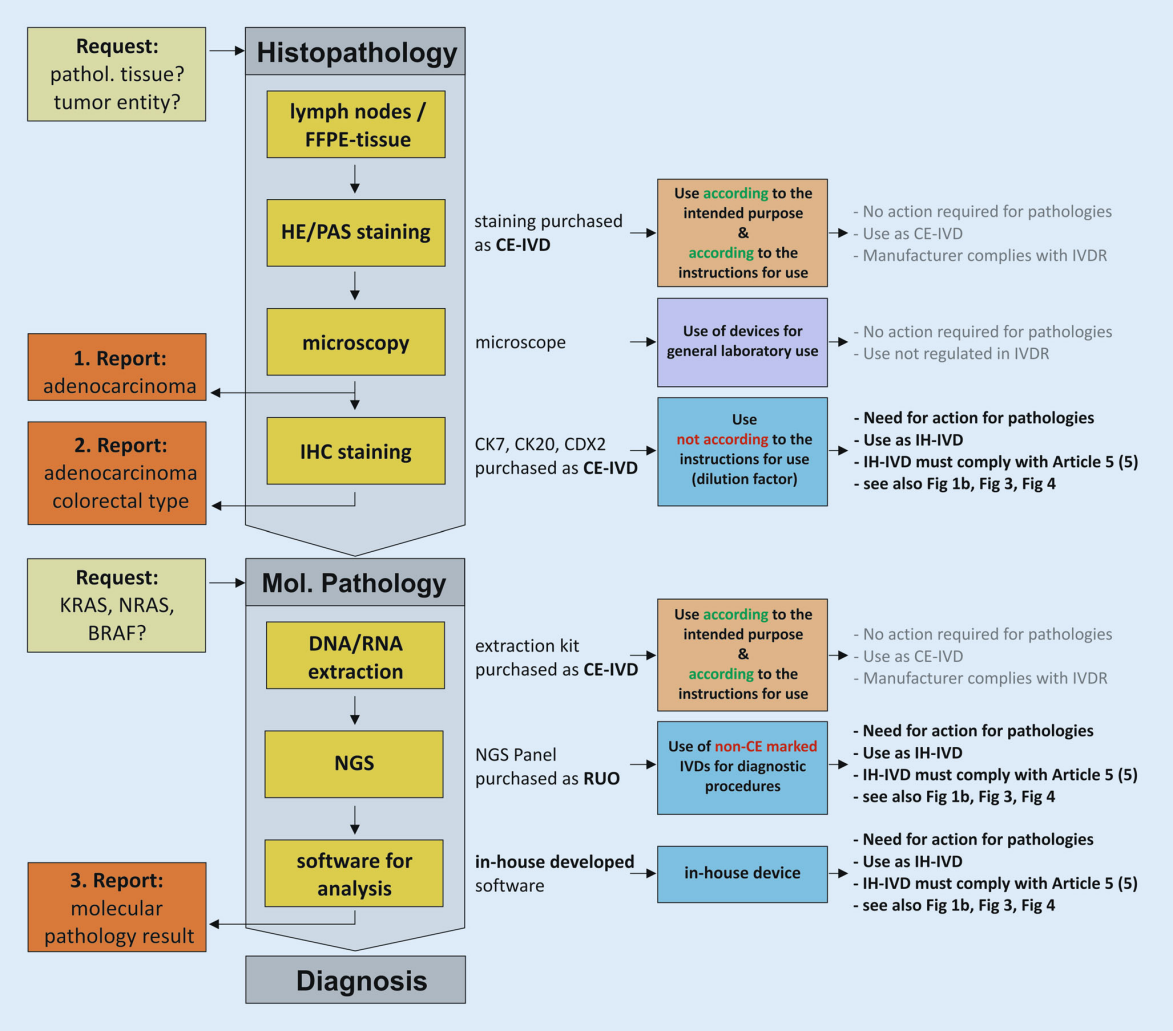
Exemplary process chain within pathology institutes from request to reporting to diagnosis using various CE-marked (*CE-IVD*) and in-house in vitro diagnostic devices (*IH-IVD*). *FFPE* formalin-fixed paraffin-embedded, *IHC* immunohistochemistry, *NGS* next-generation sequencing, *RUO* research use only, *IVDR* regulation on in vitro diagnostic devices

Within a defined process chain from specimen collection or receipt to diagnostic findings, different types of in vitro diagnostic devices can be used (Fig. 1a). Here, both commercial devices (CE-IVDs, RUO devices) and IH-IVDs are used or combined with one another (Fig. 1b). Here, the complementary or combined use of industrially manufactured IVDs, in-house procedures, and materials of general laboratory use leads to a valid finding, which is necessary for optimal patient care (example in Fig. 2). The IVDR now not only regulates the manufacture and placing on the market of industrially manufactured in vitro diagnostic devices but also imposes conditions on the manufacture and use of IH-IVDs for internal use by health institutions. This includes the use of RUO devices (Fig. 1b and 2) in diagnostics. The definition of “in vitro diagnostic medical device” has been slightly modified by the new IVDR and now explicitly covers stand-alone software (Table 1). However, a diagnostic procedure itself does not constitute an in vitro diagnostic device according to the definition of the IVDR.

The new and stricter requirements for manufacturers to obtain approval for their IVDs and the lack of grandfathering for products that have already been approved are likely to have a direct impact on price developments. Devices that are relevant for patients but become uneconomical due to the increased requirements could even be withdrawn from the market altogether. For example, important but rarely used tests for patients with rare diseases could no longer be performed or would have to be compensated by self-developed tests. In July 2021, a survey of 115 manufacturers for the European market conducted by MedTech Europe, the European trade association representing the medical technology industries, revealed that they are unlikely to transfer all CE-IVD devices to the new regulation [20, 28]. This would affect approximately one in five CE-IVDs currently available on the market, which would then no longer be available to health institutions for patient care or would have to be replaced by an in-house developed and validated IH-IVD. Devices stocked by health institutions may continue to be used until their expiration date [10].

According to recital 29 of the preamble to the IVDR, the development, manufacture, and use of in-house tests by health institutions should continue to be an option for addressing the specific needs of patients. This should continue to be possible without the involvement of conformity assessment bodies—the so-called notified bodies—and without the exclusive use of CE-marked devices. However, this regulation now aims to clarify and tighten the rules for IH-IVDs to ensure the “*highest level of health protection*” (recital 28). These recitals are specified in Article 5 (5) of the IVDR. This article imposes several conditions on health institutions for the manufacture and use of IH-IVDs. It also describes that all other requirements of the IVDR shall not apply to health institutions once the conditions set out therein have been met.

## Use of in-house in vitro diagnostic medical devices in health institutions—Article 5 (5)

**Fig. 3:**
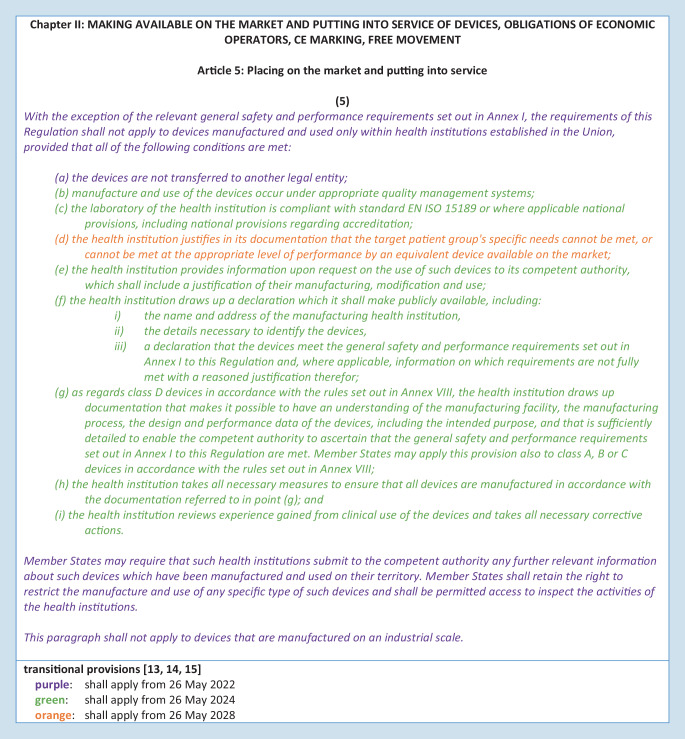
Regulation 2017/746 on in vitro diagnostic devices (IVDR)—Article 5 (5) [31]

For optimal patient care, internally developed, optimized, and established IH-IVDs are used in departments of pathology and other health institutions (e.g., PCR-based analyses or immunohistochemical tests). Article 5 of the IVDR generally regulates the placing on the market and putting into service of in vitro diagnostic medical devices and specifically regulates in paragraph 5—in contrast to the previous IVD Directive 98/79—also in-house devices (IH-IVDs), which are “*manufactured and used only within health institutions established in the Union*” and are not manufactured on an industrial scale (Fig. 3). Thus, the introduction of the IVDR also has a major impact on health institutions that use RUO devices and adapted or in-house developed devices complementarily to CE-marked IVDs. For such in-house devices, the requirements of the IVDR do not apply provided that several conditions (a)–(i) defined in Article 5 (5) and the relevant general safety and performance requirements defined in Annex I are met.

### Article 5 (5)—condition a)

***“a) the devices are not transferred to another legal entity”***

Condition a) restricts the transfer of an internally developed IVD device. In-house devices may not be transferred to another legal entity. Although the physical devices themselves may not be transferred, this restriction does not affect the transfer or publication of development or testing protocols, documentation, or results necessary to maintain high quality in health institutions through professional exchange, interpretation, and scientific discussion.

### Article 5 (5)—condition b)

***“b) manufacture and use of the devices occur under appropriate quality management systems”***

The manufacture and use of in-house devices in health institutions must occur “*under appropriate quality management systems.*” The “*appropriate quality management system*” is not conclusively defined by the IVDR; thus, it does not specify how a quality management system appropriate for the manufacture and use of IH-IVDs is defined for health institutions that manufacture IH-IVDs. Article 10 (8) can provide some guidance in this context. This article lists a minimum of aspects and criteria that a quality management system for manufacturers must consider and include. However, and importantly, Article 5 (5) excludes the application of all requirements of the IVDR for in-house devices in case of compliance with the conditions listed within Article 5 (5). Thus, the requirements of Article 10 (8) address economic operators and the manufacture of commercial IVDs and not health institutions with their manufacture of IH-IVDs or their use of RUO devices under Article 5 (5). The “*appropriate quality management system*” required for health institutions for the manufacture and use of in-house IVDs therefore leaves scope of action and room for interpretation.

### Article 5 (5)—condition c)

***“c) the laboratory of the health institution is compliant with standard EN ISO 15189 or where applicable national provisions, including national provisions regarding accreditation”***

Condition c) in Article 5 (5) concretizes this scope of action for the laboratories of the health institutions in such a way that it designates the EN ISO 15189 standard or, if applicable, national regulations including national accreditation regulations. However, condition c) differs from the above-mentioned condition b) in that condition c) focuses on the laboratory of the health institution itself and not on the device with its manufacture and use. The EN ISO standard explicitly mentioned in condition c) is not to be seen as exclusive, but rather as an exemplary possibility of design. The EN ISO 15189 standard describes the requirements for quality and competence of medical laboratories. It depicts the requirements for the quality management system as well as the general requirements for laboratory operations. A comparative analysis of the requirements from Article 5 (5) with the EN ISO 15189 standard has shown only partial conformity with the IVDR for the manufacture of in-house IVDs [24]. Due to the strong focus on the IVD device itself, the requirements of the IVDR set out in Article 5 (5) go beyond the EN ISO 15189 standard.

Although a quality management system complying with this standard is a prerequisite, accreditation is not explicitly required. In Germany, many departments of pathology comply with the standard DIN EN ISO/IEC 17020 or are accredited as so-called inspection bodies according to this standard. The German accreditation body DAkkS (Deutsche Akkreditierungsstelle GmbH) describes this aspect on its homepage: “*The DIN EN ISO/IEC 17020 standard is relevant for the accreditations of the services offered in the field of pathology in patient care. All conformity assessment bodies in pathology are thus inspection bodies. The accreditation focuses on the expert assessment of the physician—on the diagnosis. The requirements of the ISO 15189 standard are also taken into account according to ILAC P15:07/2016*” ([8], translation from German into English by the authors). Hereby, the accreditation body DAkkS emphasizes that the standard DIN EN ISO/IEC 15189 mentioned in the IVDR plays a special role in the field of pathology in Germany. Therefore, DAkkS also includes this standard in the assessment and accreditation of departments of pathology according to DIN EN ISO/IEC 17020.

It should therefore be noted that departments of pathology accredited to DIN EN ISO/IEC 15189 or DIN EN ISO/IEC 17020 meet condition c) of the IVDR. Facilities without accreditation are not required to be accredited to comply with the IVDR but must operate an appropriate quality management system.

### Article 5 (5)—condition d)

***“d) the health institution justifies in its documentation that the target patient group’s specific needs cannot be met, or cannot be met at the appropriate level of performance by an equivalent device available on the market”***

Health institutions must provide justified documentation that “*the target patient group’s specific needs cannot be met, or cannot be met at the appropriate level of performance by an equivalent device available on the market,*” as stated in Article 5 (5)—condition d). The opening of the IVDR to health institutions by Article 5 (5) is severely curtailed in this condition by elevating commercial market products compared to in-house devices. Thus, in condition d), the IVDR privileges commercial IVD manufacturers by prohibiting the diagnostic use of in-house devices if an equivalent device is available on the market. If equivalent, the commercial device must be used. Of note, this condition (so-called industry privilege) represents a fundamental paradigm shift, as the standard of quality is no longer dominated by the academic-scientific side, but by industry standards. The “*equivalent device available on the market*” is not further defined. It is unclear whether a like CE device or even an RUO device precludes the diagnostic use of an IH-IVD.

This industry privilege implicitly requires health institutions that develop, establish, and use in-house devices to allocate additional time and human resources and represents an increased documentary burden. This requires a precise definition of the IH-IVD (intended purpose) and its areas of application (type of underlying tissue, sample size, etc.) that ensures comparability and emphasizes potential superiority over CE-IVDs. The intended purpose forms the basis for finding equivalence of CE-marked devices. Devices can only be “*equivalent*” if they have the same intended purpose. Since the terms “*equivalent*” and “*the target patient group’s specific needs*” are not defined, there is room for interpretation, which should be used adequately by diagnostic colleagues and professional associations.

The assessment of equivalence must be updated by means of documented and regular, but temporally undefined, market monitoring. As a result, an elaborately developed in-house test could very quickly lose its authorization for use, possibly already during the development process, as soon as an equivalent CE-IVD reaches market maturity. However, health institutions need not fear competitive lawsuits from manufacturers and distributors of commercial CE-IVDs as a result of this requirement, as they each serve different markets. Since the regularity of the equivalence assessment is not defined in the IVDR, individual intervals adapted to the devices can be defined and specified in the risk management plan.

The IVDR requires economic operators to register their CE-IVDs in detail in the European Database on Medical Devices (EUDAMED). EUDAMED aims to improve market surveillance by mapping the lifecycle of medical devices (and thus IVDs) in real time [9]. Thus, this database also assists health institutions in fulfilling condition d). However, health institutions themselves do not have to use the database.

The IVDR also does not explicitly describe how to proceed in the event of a potentially necessary, rapid, and short-term compensation of a CE-IVD by an IH-IVD in the event of supply or production bottlenecks, but this scenario would be a case of nonavailability with the regulations intended for this purpose.

### Article 5 (5)—condition e)

***“e) the health institution provides information upon request on the use of such devices to its competent authority, which shall include a justification of their manufacturing, modification, and use”***

**Fig. 4:**
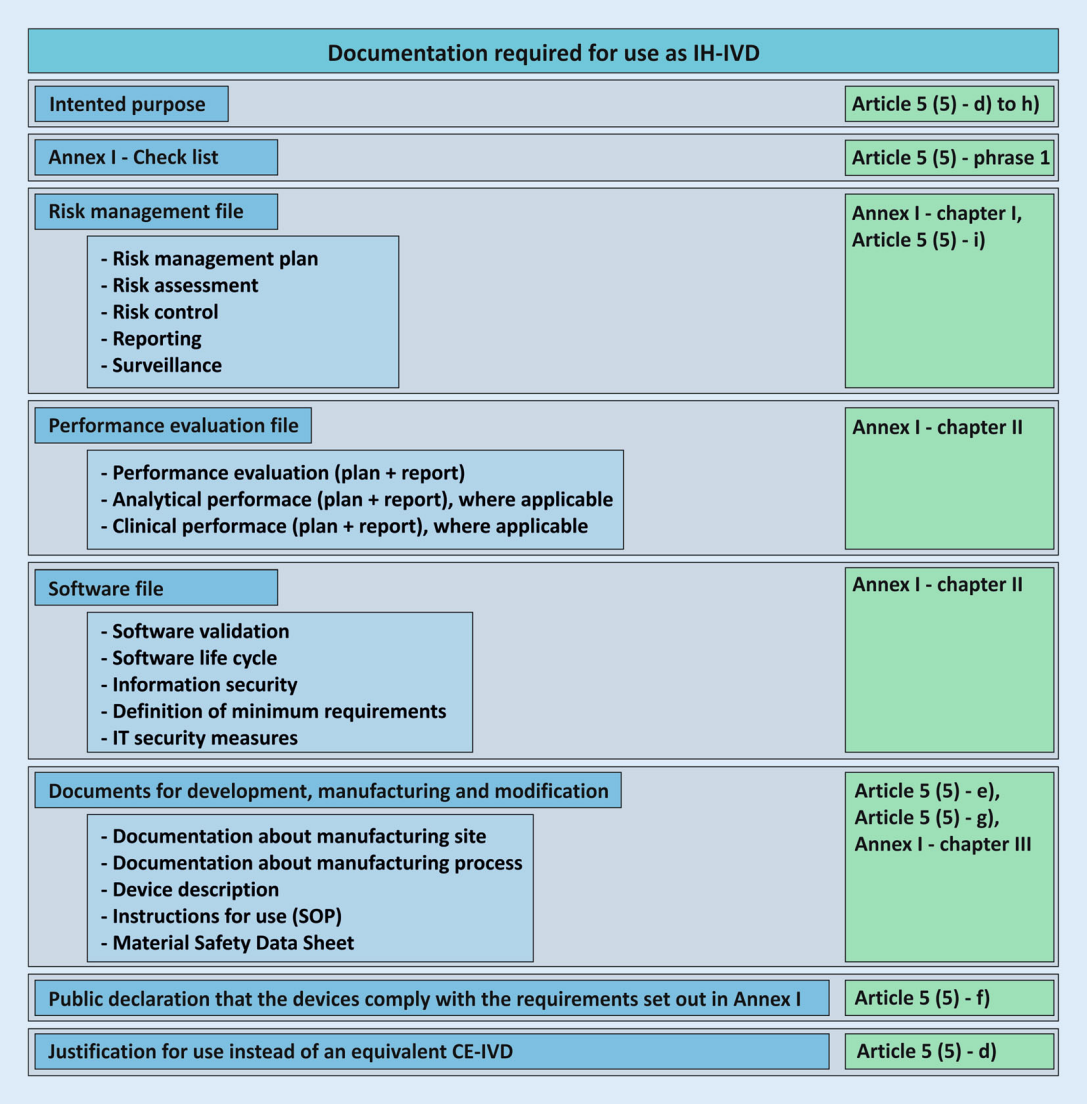
Required documentation for the use of an in-house in vitro diagnostic device (*IH-IVD*) in health institutions with reference to the regulation on in vitro diagnostic devices (IVDR; *green*). These documents do not necessarily apply to all devices and may not be complete. *SOP *standard operating procedure

The documentation resulting from Article 5 (5) for the development and manufacture of in-house devices must be made available to the competent authority upon request (see also conditions g) and h); Fig. 4). Written documented information and justifications for the use, manufacture, and modification of the devices must be available for this purpose and must be available for review by the competent state authority. In Germany, the responsibility lies with the respective responsible regional state authority. It is important to note at this point that the competent authority does not correspond to the notified bodies, which are responsible for the conformity assessment of the devices of economic operators according to Article 48 with Annexes IX to XI.

### Article 5 (5)—condition f)

***“f) the health institution draws up a declaration which it shall make publicly available, including:***

***(i) the name and address of the manufacturing health institution,***

***(ii) the details necessary to identify the devices,***

***(iii) a declaration that the devices meet the general safety and performance requirements set out in Annex I to this Regulation and, where applicable, information on which requirements are not fully met with a reasoned justification therefor”***

Health institutions that manufacture in-house devices must issue a declaration similar to the former *Medizinprodukte-Verordnung* (German Medical Devices Regulation; MPV, § 5 [6]) that identifies them as a health institution (subitem i) and the respective device as such (subitem ii). In addition, the health institution declares in writing the conformity of the devices with the essential safety and performance requirements according to Annex I of the IVDR (subitem iii). Annex I requirements that cannot be met must be justified. These declarations must be made publicly available. The IVDR does not give a more precise indication of public accessibility; so, for example, an indication on the homepage would be possible.

### Article 5 (5)—conditions g) and h)

***“g) as regards class D devices in accordance with the rules set out in Annex VIII, the health institution draws up documentation that makes it possible to have an understanding of the manufacturing facility, the manufacturing process, the design and performance data of the devices, including the intended purpose, and that is sufficiently detailed to enable the competent authority to ascertain that the general safety and performance requirements set out in Annex I to this Regulation are met. Member States may apply this provision also to class A, B or C devices in accordance with the rules set out in Annex VIII”***

***“h) the health institution takes all necessary measures to ensure that all devices are manufactured in accordance with the documentation referred to in point (g)”***

In Annex VIII the IVDR describes seven rules for manufacturers to classify IVDs into four classes of increasing individual and public risk (A to D) based on their intended purpose.

Health institutions thus classify their IH-IVDs based on their self-defined intended purpose. Most IH-IVDs used in pathology departments can be classified as class C (includes, e.g., devices used for cancer diagnosis or detection of an infectious agent with high individual but moderate public risk), class B (includes, e.g., devices that are controls with no qualitative or quantitative value or products that cannot be classified as classes A, C, and D), or class A (includes, e.g., devices for general laboratory use such as buffer solutions or histological stains).

Condition g) of Article 5 (5) tightens the documentation for devices classified in the highest risk class D (rules 1 and 2). Here, the documentation requirements listed in conditions d)–f) are extended and supplementary and more detailed documentation is required. The extended documentation enables the authority to ensure that the essential safety and performance requirements according to Annex I are fulfilled. EU member states may also extend the more stringent documentation requirement to in vitro diagnostic medical devices that are classified as class A, B, or C. The German legislator has done so, although the authorized Federal Ministry of Health has not yet made use of this possibility.

On the one hand, condition h) covers “*all devices,*” i.e., also those of classes A, B, or C, but on the other hand, it refers to the documents mentioned in condition g). However, as the Union legislator has obliged health institutions in condition g) only with regard to IH-IVDs of class D to prepare the documentation referred to therein, condition h) cannot result in an indirectly tightened documentation obligation also being constituted for in vitro diagnostic medical devices of classes A, B, or C, as long as the legislator does not extend this obligation to such devices as well. Condition h) is therefore only relevant for IH-IVDs classified in class D.

### Article 5 (5)—condition i)

***“i) the health institution reviews experience gained from clinical use of the devices and takes all necessary corrective actions”***

Condition (i) requires health institutions to conduct a review of the clinical use experience of their IH-IVDs. Consequently, resulting necessary corrective actions must be derived and taken. This systematic process was already mandatory for health institutions by the former German *Medizinprodukte-Verordnung* (MPV, § 5 [6]). The process as such is also part of the standards DIN EN ISO/IEC 15189 for medical laboratories and DIN EN ISO/IEC 17020 for institutes of pathology.

## Safety and performance requirements—Annex I

**Fig. 5:**
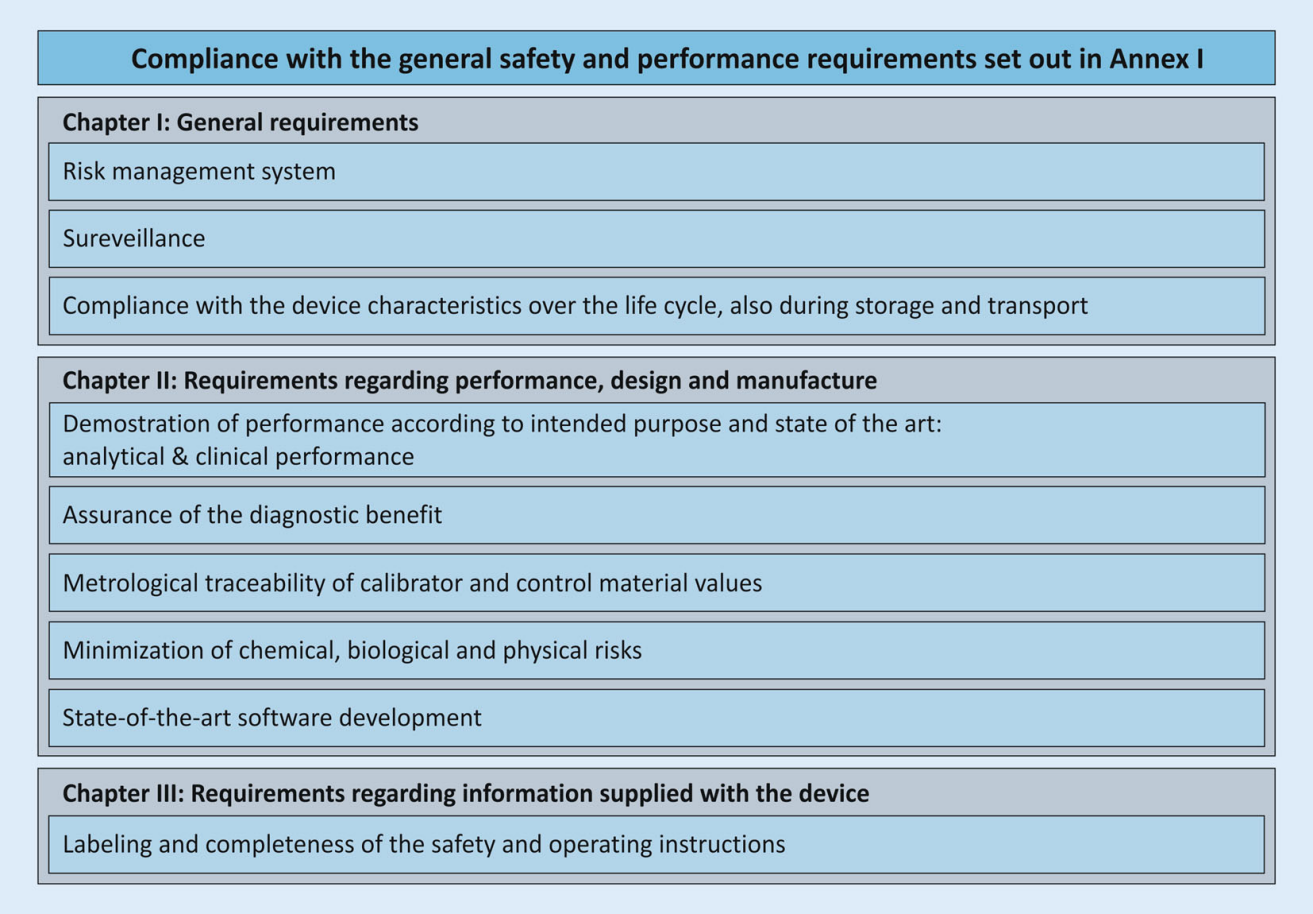
Elements from Annex I, which are necessary for the fulfillment of the essential safety and performance requirements. These elements do not necessarily apply to all devices and may not be complete

As of May 26, 2022, IH-IVDs of health institutions must comply with the general safety and performance requirements (Annex I of the IVDR) according to Article 5 (5). This Annex is divided into three chapters: (I) general requirements; (II) requirements regarding performance, design, and manufacturing; (III) requirements regarding information supplied with the device (Fig. 5). The purpose is to ensure and demonstrate that the devices (1) are fit for their intended purpose, that they (2) are safe and effective, and that they (3) do not endanger the health of users, third parties, or patients, and that any potential risk is under control and acceptable.

This annex describes the general safety and performance requirements for all IVD devices and applies to CE-IVDs placed on the market as well as to IH-IVDs of health institutions. However, not all requirements are always applicable to IH-IVDs, and the wording suggests that Annex I focuses more on economic operators and commercial devices. Health institutions should consider which requirements specifically apply to their IH-IVDs and create a checklist for their needs that will guide them through the annex. Condition f) of Article 5 (5) requires a publicly available statement from the health institution that its IH-IVDs comply with the requirements of Annex I. For devices that may be classified as class D under Annex VIII, condition g) of Article 5 (5) requires much more extensive documentation for this purpose.

## Risk management and safety evaluation

An essential core point of Annex I is the definition and ongoing execution of a risk management system. This product-specific risk management system required by the IVDR is only partially covered by the ISO 15189 standard mentioned under condition c) and the systemic risk management system described there [24]. This notion also applies to the standard DIN EN ISO/IEC 17020. However, these standards provide a basis for a primarily institutional and general risk management system, which can be extended for the manufacture and use of IH-IVDs. The two standards ISO 22367 and ISO 14971, which specify a risk management process for medical laboratories, can provide assistance in this regard. The content of the ISO 22367 standard also covers, for example, the manufacture and use of in-house IVDs. According to the IVDR, a risk management system is only required for those diagnostic laboratory tests in which in-house devices are used.

Annex I requires health institutions to perform a comprehensive safety assessment for each IH-IVD to assess and minimize risks to the user, the patient, or, if applicable, third parties (in this case, trained laboratory personnel). It is important not only to identify risks, but also to derive their probabilities and the subsequent measures to avoid or control risks. Since the risks of the methodologies (polymerase chain reaction [PCR], real-time PCR [qPCR], next-generation sequencing [NGS], etc.) are likely to be similar irrespective of the exact molecular target, the development of in-house templates is possible here.

## Performance evaluation

Annex I, chapter II, paragraph 9 contains the performance requirements for IVD devices to ensure that the devices are fit for their intended purpose. Here, the regulation lists performance parameters for analytical performance and clinical performance that are to be provided “*where applicable*” for the device. How such validation is to be performed is to be determined for each device and according to the generally accepted state of the art. In Article 56 and Annex XIII, the IVDR itself describes how a performance evaluation is to be performed for CE-IVDs to be placed on the market. These specifications can also be used as a reference for IH-IVDs. The standard DIN EN ISO 15189, which health institutions must comply with according to condition c) of Article 5 (5), the standard DIN EN ISO/IEC 17020, and the guideline for quality assurance of laboratory medical examinations issued by the German Federal Medical Association (*Richtlinie der Bundesärztekammer zur Qualitätssicherung laboratoriumsmedizinischer Untersuchungen*—Rili-BÄK, [22]), also require that all procedures must be fully validated before being introduced into routine diagnostics [7]. In Germany, guidance at the national level is provided by the guidelines of the DAkkS Pathology/Neuropathology Sector Committee, which is responsible for interpreting accreditation requirements in the field of pathology. They include guidance for validations and verifications of sub-procedures in PCR-based molecular pathology [6] and immunohistology [5]. Across Europe, the Biomedical Alliance Europe is currently developing more detailed guidance on how to implement the IVDR for IH-IVDs as well [3]. In addition, for the field of NGS-based molecular pathology, reference can be made to the U.S. Food and Drug Administration (FDA) guidelines [17] and scientific publications [18, 26], which reflect the state of the art and can serve as a template for an individual and device-specific validation plan. These documents provide technical and scientific guidance on how to meet the requirements for validation and verification of molecular pathology or immunohistochemistry assays. They also describe practical examples of implementation. For example, the comparison with commercial standards, external reference standards, and with orthologous validated or commercial methods is presented. For the verification of the correctness of IH-IVDs, e.g., (commercial) control samples or participation in interlaboratory comparisons are suitable. For further design and definition of the performance evaluation, the expertise of pathologists and the respective professional societies and working groups is essential. The important role of networks, in which, for example, validation strategies, protocols, and control samples can be exchanged, should also be emphasized.

## Minimum requirements of an intended purpose

The intended purpose, Annex I, chapter III, 20.4.1 c), focuses on a clear definition of the device, the processes, its capabilities, and areas of application. It is used as a benchmark for evaluating the similarity or superiority of an IH-IVD over a CE-IVD. Also, the intended purpose specifies the scope of performance requirements. A precise definition of the clinical diagnostic question (testing for pathogenic changes, determination of tumor entity, genetic change sought, pathogen detection, etc.), the material used (formalin-fixed paraffin-embedded [FFPE], fresh material, amount of material used, etc.), and the metrics of the methodology used from the performance evaluation creates a concrete evaluation matrix with which different IVDs must be measured. The purpose, therefore, represents an important part of the decision regarding whether the establishment of an IH-IVD is necessary or whether a comparable CE-IVD must be resorted to.

## Discussion and conclusion

The primary intention and rationale of the IVDR is to control the quality of diagnostic devices from commercial manufacturers to improve patient safety. In general, this intention is to be welcomed. Exemplifying that this stricter regulation can be necessary, a recent study by the Paul Ehrlich Institute in collaboration with researchers from other institutions reviewed severe acute respiratory syndrome coronavirus 2 (SARS-CoV-2) rapid antigen tests available in Europe and found poor sensitivity in about 21% [23]. Currently, manufacturers are still allowed to CE mark these tests themselves as IVDs (so-called self-certification) and place them on the market. In the future, an EU reference laboratory and a notified body will be required for certification, as these rapid tests fall into the highest risk class D according to the IVDR.

At the same time, the IVDR recognizes the fact of in-house developed tests (opening clause) that are used by health institutions. While this aspect is generally positive, the IVDR, via the industry privilege described above, inherently completes a paradigm shift from the previous quality specification by (non-industrial) academic experts to a quality benchmark according to industry standards. Health institutions provide diagnostic services to patients and represent them in terms of content as well as legal aspects. It is therefore of great importance that the above-mentioned points of Article 5 (5) are addressed and substantially shaped by stakeholders of the health institutions. The IVDR is intended to achieve standardization and quality monitoring without using the analogue of the Food and Drug Administration (FDA; for CE-IVDs) and Clinical Laboratory Improvement Amendments (CLIA; for IH-IVDs) on the national and European executive side, which are both large institutions that review and monitor the marketing and use of diagnostic tests in the US. The IVDR, therefore, delegates these tasks to notified bodies (for CE-IVDs) on the one hand and to (local) state authorities (for IH-IVDs) on the other. This constellation will likely lead to great heterogeneity and bottlenecks across the EU. In addition, the massively increased demand and simultaneous shortage of notified bodies pose great challenges for economic actors. There is concern that established CE-IVDs could be taken off the market, prices could increase, or new IVDs could only come onto the market as RUO devices.

Articles 98 and 99 of the IVDR describe and define the tasks of the Medical Device Coordination Group (MDCG). One of its tasks is the development of guidelines for the harmonized implementation of the IVDR in order to support stakeholders in the application of the regulation. A guidance document specific to IH-IVDs and their use by health institutions was published in early 2023 [11]. These guidance documents are not legally binding but represent a blueprint for the practical application of the IVDR to achieve effective and harmonized implementation of the regulations. Assistance for the implementation of the IVDR is provided by templates, checklists, and handouts of the Ad-Hoc Commission In-Vitro Diagnostics of the German Working Group of Scientific Medical Societies (Arbeitsgemeinschaft der Wissenschaftlichen Medizinischen Fachgesellschaften e. V., AWMF), which are freely available for use and modification [1].

Due to the definitional gaps and vagueness of the IVDR outlined above, there is also an opportunity for professional societies and associations to contribute their academic knowledge and expertise to the content of the IVDR, e.g., the AWMF [1], German Society for Pathology [25], Federal Association of German Pathologists [4], Dutch Society of Pathology [2, 27], European Hematology Association [19], or the Biomedical Alliance Europe [3]). Only through this approach can a successful application and implementation of the IVDR succeed and substantial gaps in supply and inadequate pricing of diagnostics be avoided. In this context, IH-IVDs will continue to contribute to precise diagnostics and optimal patient care.

## Conclusion for practice

- Regulation (EU) 2017/746 on in vitro diagnostic medical devices (IVDR) entered into force on May 26, 2017.
- The new amending regulation, dated December 15, 2021, defines a phased introduction of the IVDR, for both commercial CE-IVDs and in-house IVDs (IH-IVDs) from health institutions.
- The primary intention of the IVDR, namely to improve patient safety, is to be welcomed. Thus, EU-wide harmonized requirements are intended to improve the quality control and safety of diagnostic devices.
- In this context, the IVDR recognizes the benefit and necessity of in-house developed tests (opening clause) used by health institutions.
- The IVDR is primarily aimed at manufacturers of IVDs, but also has a drastic impact on pathology institutes.
- The IVDR imposes several conditions on health institutions that develop and use in-house IVDs.
- Due to definitional blanks and vagueness within the IVDR, there is an opportunity for professional societies and associations to contribute their academic knowledge and expertise to the content design of the IVDR and to shape the IVDR to fulfill its purpose.

## Declarations

For this article no studies with human participants or animals were performed by any of the authors. All studies mentioned were in accordance with the ethical standards indicated in each case.

The supplement containing this article is not sponsored by industry.
